# Impact of MoS_2_ Monolayers on the Thermoelastic Response of Silicon Heterostructures

**DOI:** 10.1021/acsanm.4c02096

**Published:** 2024-07-01

**Authors:** Davide Soranzio, Denny Puntel, Manuel Tuniz, Paulina E. Majchrzak, Alessandra Milloch, Nicholas M. Olsen, Wibke Bronsch, Bjarke S. Jessen, Danny Fainozzi, Jacopo S. Pelli Cresi, Dario De Angelis, Laura Foglia, Riccardo Mincigrucci, Xiaoyang Zhu, Cory R. Dean, Søren Ulstrup, Francesco Banfi, Claudio Giannetti, Fulvio Parmigiani, Filippo Bencivenga, Federico Cilento

**Affiliations:** † Institute for Quantum Electronics, Eidgenössische Technische Hochschule (ETH) Zürich, CH-8093 Zurich, Switzerland; ‡ Dipartimento di Fisica, Università degli Studi di Trieste, IT-34127 Trieste, Italy; § Department of Physics and Astronomy, Interdisciplinary Nanoscience Center (iNANO), Aarhus University, 8000 Aarhus C, Denmark; ∥ Department of Mathematics and Physics, Università Cattolica del Sacro Cuore, IT-25133 Brescia, Italy; ⊥ ILAMP (Interdisciplinary Laboratories for Advanced Materials Physics), Università Cattolica del Sacro Cuore, IT-25133 Brescia, Italy; # Department of Physics and Astronomy, KU Leuven, B-3001 Leuven, Belgium; ¶ Department of Chemistry, Columbia University, New York, New York NY-10027, United States; ∇ Elettra—Sincrotrone Trieste S.C.p.A., Strada Statale 14, km 163.5, IT-34149 Trieste, Italy; ○ Department of Physics, Columbia University, New York, New York 10027, United States; ⧫ Université de Lyon, CNRS, Université Claude Bernard Lyon 1, Institut Lumière Matière, F-69622 Villeurbanne, France; †† CNR-INO (National Institute of Optics), IT-25123 Brescia, Italy; ‡‡ International Faculty, University of Cologne, Albertus-Magnus-Platz, D-50923 Cologne, Germany

**Keywords:** MoS_2_, monolayer, time-resolved, thermoelasticity, heterostructure, SAW, dynamics

## Abstract

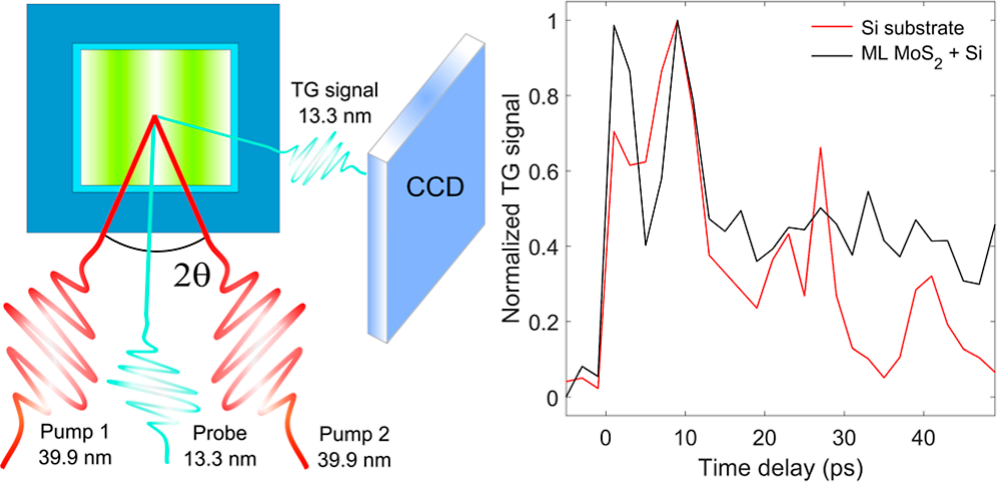

Understanding the thermoelastic response of a nanostructure is crucial for the choice of materials and interfaces in electronic devices with improved and tailored transport properties at the nanoscale. Here, we show how the deposition of a MoS_2_ monolayer can strongly modify the nanoscale thermoelastic dynamics of silicon substrates close to their interface. We demonstrate this by creating a transient grating with extreme ultraviolet light, using ultrashort free-electron laser pulses, whose ≈84 nm period is comparable to the size of elements typically used in nanodevices, such as electric contacts and nanowires. The thermoelastic response, featuring coherent acoustic waves and incoherent relaxation, is tangibly modified by the presence of monolayer MoS_2_. Namely, we observed a major reduction of the amplitude of the surface mode, which is almost suppressed, while the longitudinal mode is basically unperturbed, aside from a faster decay of the acoustic modulations. We interpret this behavior as a selective modification of the surface elasticity, and we discuss the conditions to observe such effect, which may be of immediate relevance for the design of Si-based nanoscale devices.

## 1 Introduction

Transition metal dichalcogenides (TMDs) are a class of materials composed of atomic layers held together by van der Waals interactions, much weaker than the intralayer covalent bonds, giving them a marked two-dimensional character. This feature allows to obtain controlled thicknesses down to the single layers, useful for tuning several physical properties, such as the electronic band gap, the vibrational levels, and the excitons, and for their implementation in thin nanodevices, e.g., transistors, photodetectors, and electroluminescent devices.^1−4^ In this regard, a key point is the interaction between TMDs and substrates, which has been shown to critically impact the heterostructure properties. Hence, the choice of the substrate constitutes a fundamental aspect for the application of TMDs heterostructures in electronic circuits.^5−8^ To design functional devices, the thermoelastic response of the structure, i.e., how fast can heat be dissipated, together with its elastic properties, which are also relevant for technological applications,^9,10^ are fundamental aspects to be considered.

In this work, we focus on the dynamics over the typical length-scales of components used in nanodevices, e.g., electric contacts and nanowires.^11,12^ In particular, we study the nanoscale thermoelastic response of a TMD monolayer (ML) deposited on top of a silicon substrate. The selected TMD material is molybdenum disulfide (MoS_2_), which presents an interlayer separation of 0.65 nm and a ≈1.9 eV optical band gap at the ML limit.^13^ To investigate the thermoelastic dynamics, we used the transient grating (TG) technique.^14^ ML TMDs have been the focus of previous TG experiments, although using grating periods of the order of micrometers, as obtained by infrared laser pulses.^15,16^ Differently, in our experiment, nanoscale TGs were generated on the sample using free-electron laser (FEL) extreme-ultraviolet (EUV) femtosecond pulses to attain a ≈84 nm grating period. This allows not only an investigation of phenomena on a much smaller length scale, but also a higher sensitivity to the thermoelastic response with respect to the electronic one.^17^ We studied the TG dynamics in three systems: a blank Si membrane, a ML MoS_2_ transferred on a Si membrane and a ML MoS_2_ grown on a Si wafer. The results showed a marked difference in the surface thermoelastic response. In the case of the blank membrane, two acoustic modes, a superficial Rayleigh-like wave and a longitudinal wave, were detected. In particular, the first mode makes the largest contribution to the oscillatory dynamics. Differently, the longitudinal mode dominates for the MoS_2_-covered samples alongside with a faster decay of the modulations. We ascribe this fact to the different atomic motion involved, which is elliptical and localized close to the interface for the surface mode and parallel to the interface for the longitudinal mode. Underneath the periodic modulations, we observed a slower decay of the nonoscillating background, typically linked to thermal transport,^18^ for the heterostructures compared to the blank substrate.

## 2 Experimental Section

### 2.1 Samples

**Figure 1:**
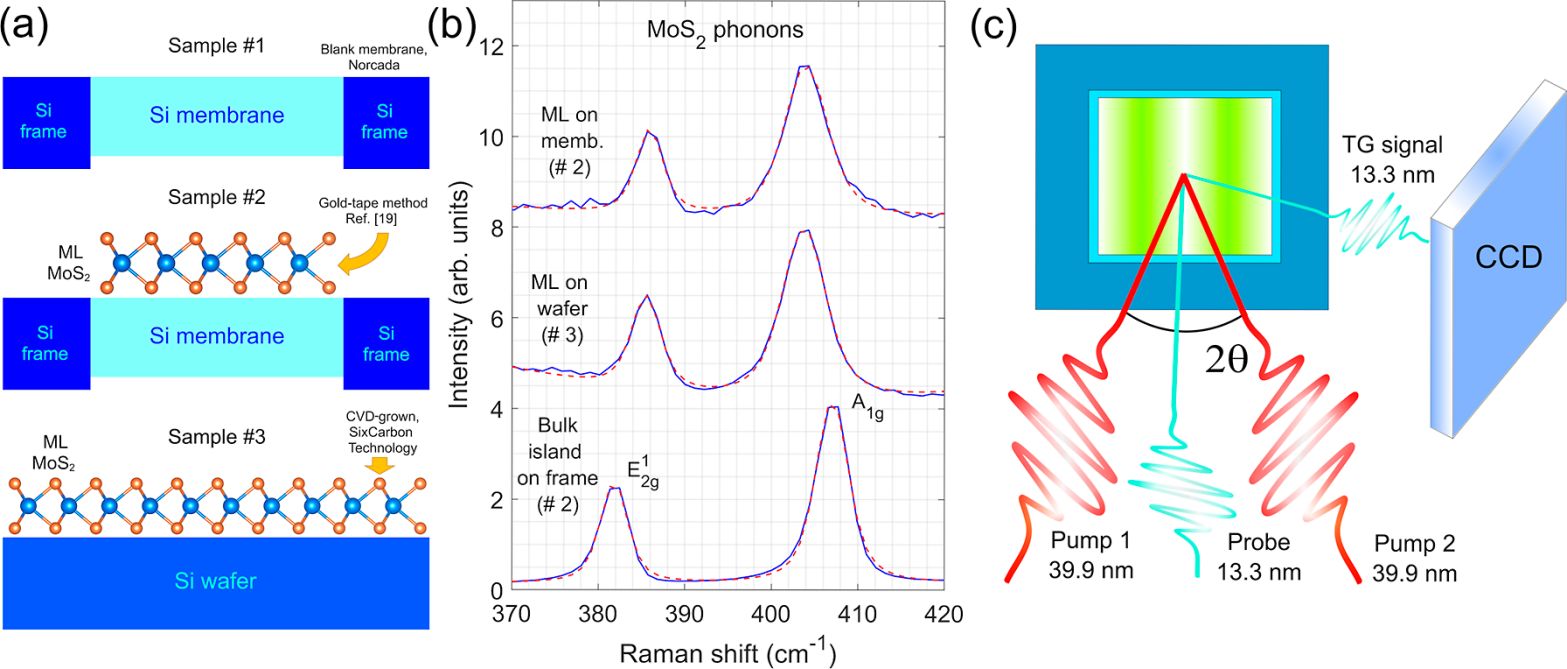
(a) Samples studied during the TG experiments: #1 blank Si membrane, #2 MoS_2_/Si membrane and #3 MoS_2_/Si wafer. (b) Selected micro-Raman spectra showing the E^1^_2g_ and A_1g_ MoS_2_ phonon peaks from the investigated samples: MoS_2_/Si membrane, MoS_2_/Si wafer and a MoS_2_ bulk island. The Raman traces were rescaled and vertically shifted for clarity. The blue lines correspond to the experimental data, while the red-dotted ones to the best fits. (c) Scheme of the TG setup at the EIS-TIMER beamline at FERMI.

Our study focused on samples based on 200 nm-thick, 1.6 × 1.6 mm^2^-large, boron-doped Si membranes, commercially produced by Norcada, nominally having <1 nm rms surface roughness on both front and back sides of the membrane, which is supported by a 200 μm-thick Si frame. The three samples are depicted in Figure 1a. One blank Si membrane was used as a reference (sample #1). On top of a nominally identical membrane, a large-area monolayer of MoS_2_ was prepared using the gold tape method^19^ (sample #2) at Columbia University. In addition to the membrane systems, we studied a CVD-grown MoS_2_ monolayer, deposited on a 0.52 mm-thick Si wafer, commercially available from SixCarbon Technology (sample #3).

### 2.2 Micro-Raman Characterization

The uniformity and thickness of the MoS_2_ films were tested with a micro-Raman Renishaw inVia instrument using a continuous-wave 532 nm laser at the I-LAMP laboratories in Brescia (Italy). Measurements were acquired by using an incident power of 5 mW for the samples with a deposited MoS_2_ monolayer and 0.5 mW for the blank substrates. At focus, under 100× magnification, the spot size is of the order of a few microns. All the measurements were performed in air. The full characterization is available in Supporting Information, Sections I and II. We took advantage of the relation between the frequencies of the MoS_2_ E^1^_2g_ and A_1g_ near-zone-center phonon modes and number of layers.^1^ Sample scans are reported in Figure 1b, with the ML-covered regions from sample #2 and #3 showing a much smaller (≈19 cm^–1^) frequency separation of the modes, when compared to a thick multilayer, bulk-like island found on the Si frame (≈25 cm^–1^), which is consistent with the literature.

### 2.3 EUV Transient Gratings

The EUV gratings were generated at the EIS-TIMER beamline^17,18,20^ using the radiation generated by the double stage FEL source (FEL2) available at the FERMI free-electron laser.^21^Figure 1c illustrates the EUV TG experimental geometry. Two symmetric pump beams with wavelength λ_pu_ = 39.9 nm and time duration < 100 fs, generated by the first stage of the FEL, cross at the sample with an angle 2θ = 27.6° generating a TG with a periodicity Π = 2π/*k*_TG_ = 83.6 nm, where *k*_TG_ = 4π sin(θ)/λ_pu_ = 0.075 nm^–1^ is the modulus of the TG vector. As a probe, we used the output of the second stage of the FEL, which was set at λ_pr_ = 13.3 nm.

The spot sizes, full width at half-maximum (fwhm), of the two pump beams were (380 × 290) and (470 × 250) μm^2^. The probe beam at the sample had a spot size of (450 × 330) μm^2^. Unlike a typical pump–probe experiment, in a TG measurement the recorded signal originates only from the probe beam fraction, which is diffracted by the transient grating. Thus, it results from only the region where the three beams are superimposed. All the measurements were performed in reflection geometry, in high vacuum, and at room temperature. The diffracted signal was recorded by using a CCD camera. The fluence values reported in the text refer to the sum of the incident fluences of the two pumps whose pulse energies are approximately equal. The FEL intensity at the sample was varied by using a nitrogen gas cell, which efficiently attenuates the radiation at λ_pu_ while it marginally affects the transmission of the probe beam. Typically, the acquisition of the EUV-TG signal takes about 1–2 min per delay point, leading to 1–2 h for a full trace and a good signal-to-noise ratio. An estimate of the uncertainty over the TG signal values at EIS-TIMER beamline was discussed by Bencivenga et al.^18^ The spatial and temporal overlap between the two pump and the probe beams were checked separately by using a reference IR laser and performing FEL-pump/IR-probe transient transmissivity experiments on a YAG reference sample.

The attenuation lengths for the incident FEL pumps in MoS_2_ and Si can be derived from the tabulated atomic data found in Henke et al.^22^ We obtain ≈40 and ≈237 nm, respectively, while these values are ≈205 and ≈583 nm at λ_pr_ = 13.3 nm. An extended wavelength dependence and comparison with other common substrates for MoS_2_ can be found in Supporting Information, Section III.

## 3 Results and Discussion

**Figure 2:**
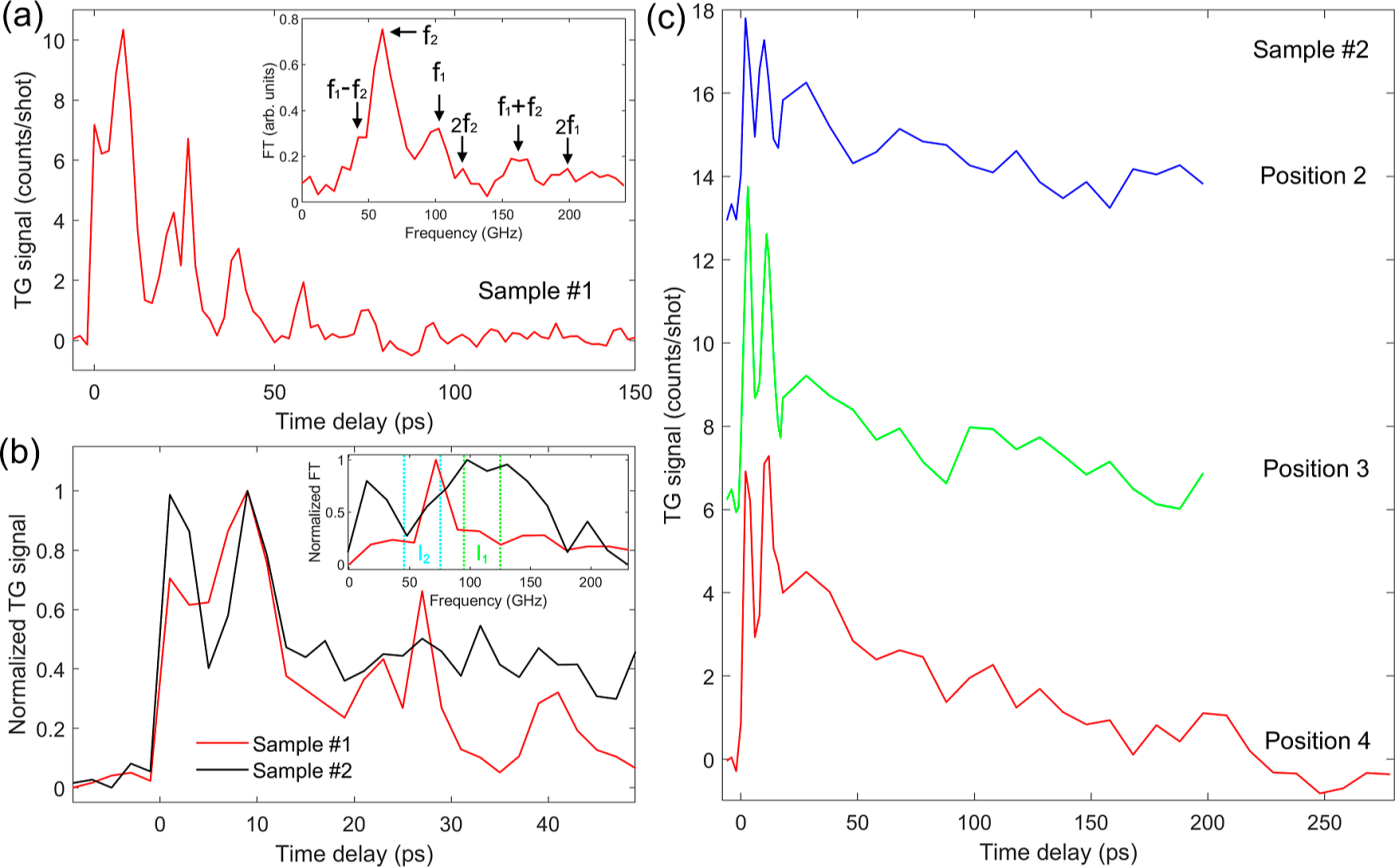
(a) TG signal from the blank Si membrane (sample #1) at 0.34 mJ/cm^2^ incident fluence; the inset shows the Fourier transform of the signal after the subtraction of an exponentially decaying background for the ≈160 ps window. (b) TG signal from the MoS_2_/Si membrane (sample #2) at 0.23 mJ/cm^2^ fluence compared to the one from (a); the inset shows the Fourier transform of the two signals after the subtraction of an exponentially decaying background for the ≈60 ps window; two integration region are marked (see text). (c) TG signal collected at different positions compared to (b) on the membrane of sample #2 under 0.25 mJ/cm^2^ fluence; the traces were shifted for clarity.

Figure 2a shows the time-resolved TG signal for the blank Si membrane (sample #1) at 0.34 mJ/cm^2^ incident fluence. After the temporal overlap between pump and probe, occurring at *t* = 0, a transient diffracted signal appears. It is characterized by modulations on top of the decay of the average signal, which is a typical thermoelastic response. To analyze the TG signal, we model its time-resolved intensity using the expression^18^
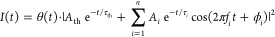
1where θ(*t*) is the Heaviside function, *A*_th_ and τ_th_ are the amplitude and time decay constant usually assigned to the thermal component, *A*_*i*_, *f*_*i*_, τ_*i*,_ and ϕ_*i*_ are the amplitude, frequency, time decay constant and phase of the *i*-th mode.

Two modes are sufficient to describe the TG signal from sample #1. In fact, the square in the formula leads, when expanded, to a series of exponentially decaying terms oscillating at frequencies 0, *f*_1_, *f*_2_, 2*f*_1_, 2*f*_2_, *f*_1_ + *f*_2_, and *f*_1_ – *f*_2_. The non-null frequency contributions can be visualized by fitting the experimental data to eq 1 with the oscillatory amplitudes *A*_*i*_ set to zero and performing the discrete Fourier transform on the residual signal (inset of Figure 2a). The result is consistent with two modes with overtones, corresponding to the sums and differences of two frequencies *f*_1_ and *f*_2_, such that *f*_1_ ≈ 102 GHz, *f*_2_ ≈ 60 GHz, 2*f*_1_ ≈ 204 GHz, 2*f*_2_ ≈ 120 GHz, *f*_1_ + *f*_2_ ≈ 162 GHz and *f*_1_ – *f*_2_ ≈ 42 GHz, with the last one appearing as the left shoulder of the most prominent peak at *f*_2_ ≈ 60 GHz. By fitting the data using the full eq 1, we obtain the time decay constant of the nonoscillating background τ_th_ = (39 ± 2) ps, the frequencies of the two modes *f*_1_ = (105 ± 1) GHz and *f*_2_ = (58.2 ± 0.4) GHz, and the time decay constants of the two modes τ_1_ = (80 ± 50) ps and τ_2_ ≈ 470 ps. These last two values are either comparable to or larger than the investigated delay range, as shown in Figure 2a, hence a more accurate estimation would require further dedicated measurements.

We can rationalize the presence of these two modes as Lamb waves, namely, acoustic waves of the membrane.^23,24^ In TG experiments, their momentum is set by *k*_TG_, while the propagation speed is related to the frequency-momentum dispersion relation of the material. Using the frequencies derived from the TG signal, it is possible to obtain the corresponding phase velocities with *v*_p,*i*_ = *f*_*i*_Π. For sample #1, the detected modes correspond to *v*_p,1_ = (8.81 ± 0.09) km/s and *v*_p,2_ = (4.87 ± 0.03) km/s. These can be compared to the predicted phase velocity dispersion for the 200 nm-thick Si membrane, through the transverse resonance method,^23,25^ and using the longitudinal and shear velocities reported in Farnell and Adler.^26^ The resulting phase velocities depend on the product ρ = *k*_TG_*b*, where, in the present case, *k*_TG_ = 0.075 nm^–1^ and *b* = 200 nm is the membrane thickness; hence ρ ≈ 15. At this ρ value, two modes with phase velocities 8.89 and 4.90 km/s, close to the longitudinal and transverse ones for Si, are predicted, compatible with the observed ones.

**Figure 3:**
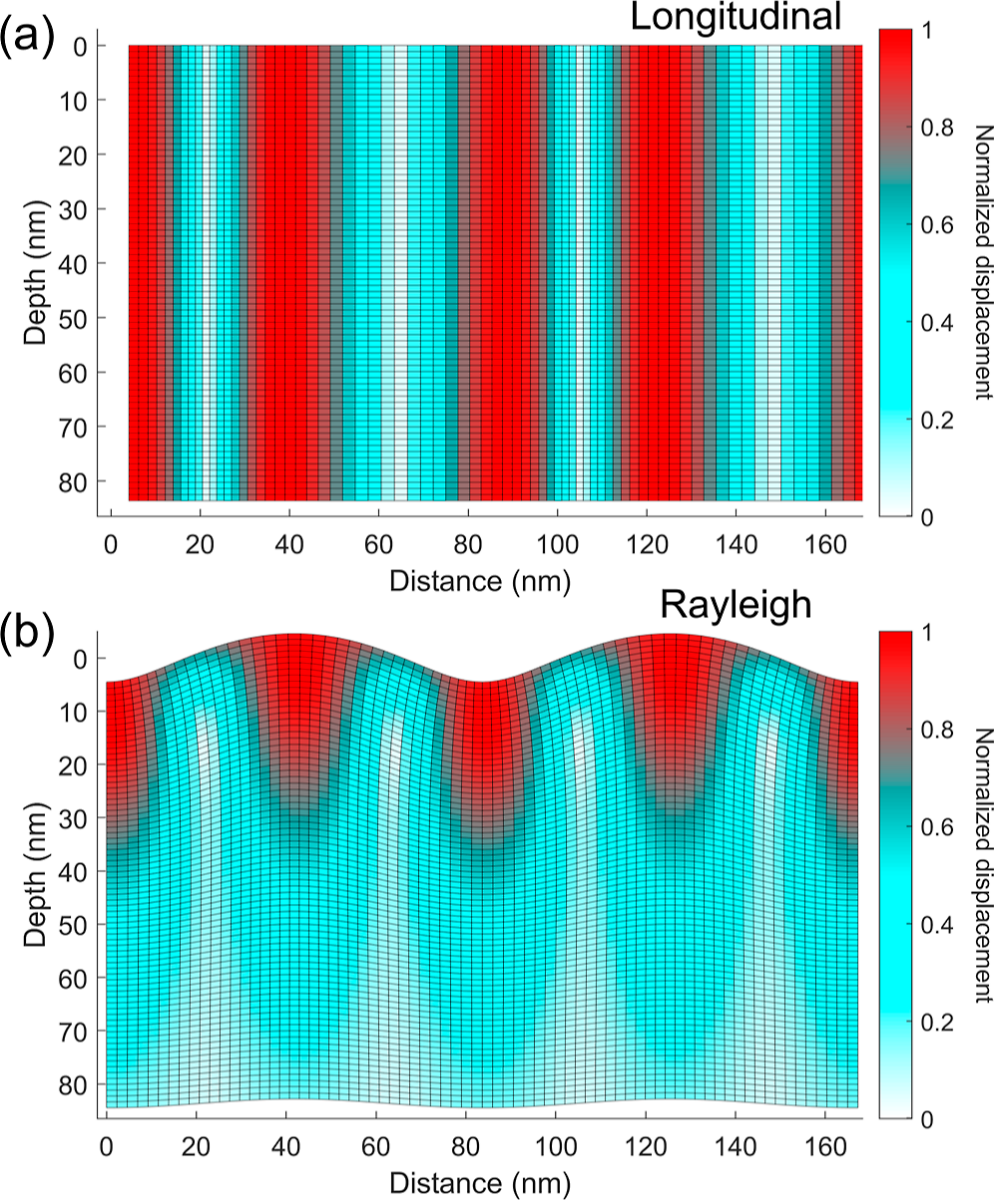
Schematic representation of the displacements for the (a) longitudinal (b) Rayleigh acoustic waves in silicon. Their amplitudes were arbitrarily set by a common scaling factor to ease visualization; in the colorbar, the displacements were normalized to the maximum one with respect to the equilibrium positions.

Similar results have been previously reported for optical and EUV TG experiments in other materials for ρ ≫ 1.^27−31^ These acoustic modes are commonly identified as the “surface-skimming” longitudinal wave (SSLW) and, in the limit of a semi-infinite substrate (ρ → ∞), the Rayleigh surface acoustic wave (RSAW). As observed in our measurements, the SSLW at *f*_1_ = 105 GHz is only visible in the first ≈15 ps in the TG signal, since it behaves like a leaky wave which rapidly decays in the bulk.^27^ The RSAW decays on a longer time scale and involves elliptical displacements, i.e., combined longitudinal and transversal motion. These are approximately localized at the surface within half a wavelength.^24,32^ A depiction of the two waves is given in Figure 3, based on the derivation of their displacements with the method of potentials;^24^ more details are provided in the Supporting Information, Section IV. The accurate prediction of wave and heat propagation is complex and depends on many experimental details, such as the energy distribution in time and space. Nevertheless, in the reflection configuration used in the experiments, one expects the dominant contribution to the TG signal to arise from a coherent surface displacement, i.e., from the RSAW.^27,33−35^

In Figure 2b we show the time-resolved TG signal for the MoS_2_/Si membrane heterostructure (sample #2) compared to the blank membrane (sample #1). Differently from the blank case, here we observe an oscillatory signal in the first ≈15 ps only, with two prominent peaks. By performing the discrete Fourier transform after subtracting the nonoscillatory signal from the two traces as for panel (a), we observe a clear difference between sample #1 and sample #2 as shown in the inset in panel (b). In fact, if we integrate the spectral amplitude around the two main mode frequencies (*f*_1_, 90–120 GHz) and (*f*_2_, 45–75 GHz), we obtain that their ratio is *I*_1_/*I*_2_ ≈ 0.6 for sample #1 and *I*_1_/*I*_2_ ≈ 1.9 for sample #2, pointing to a marked difference in the relative contribution of the two modes. Afterward, the signal gradually decays with minor modulations on a longer time scale with respect to the blank substrate (Figure 2a,c). Repeated scans at different sample positions gave an analogous response.

**Figure 4:**
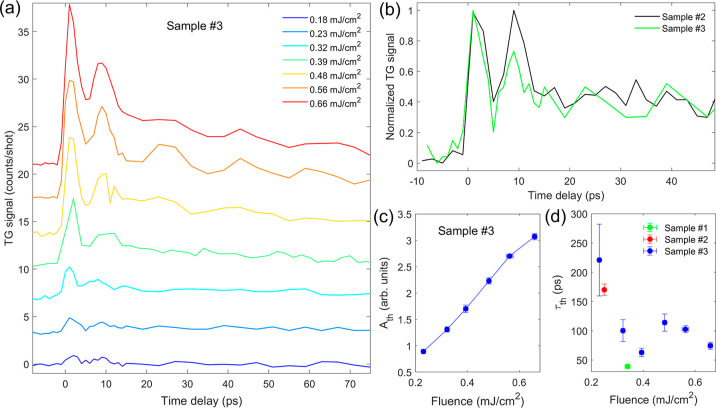
(a) Fluence dependence of TG signal from sample #3; the traces were shifted for clarity. (b) Comparison between the normalized TG signals from sample #2 (0.23 mJ/cm^2^) and sample #3 (0.25 mJ/cm^2^). (c) Amplitude and (d) time decay constant of the nonoscillating component, as derived by the best fit of EUV TG waveforms from panel (a) to eq 1. In the last panel, the results for samples #1 and #2 are reported as comparison.

Figure 4a shows a fluence dependence of the TG signal collected on the MoS_2_/Si wafer (sample #3). Analogously to sample #2, we observe a response mainly localized in the first picoseconds with two main oscillation periods, resembling the SSLW detected in sample #1, followed by lower frequency modulations that emerge only for the highest investigated fluences, similar to the ones associated with the RSAW observed for sample #1. The TG signal acquired at different positions on the sample showed analogous features, albeit with some amplitude variability possibly connected to the local MoS_2_ coverage (see Supporting Information, Section I). A comparison between the response of samples #2 and #3 under similar excitation conditions is reported in Figure 4b, showing similar features in the dynamics.

Therefore, one may expect surface modes like the RSAW, involving both longitudinal and transverse displacements,^24^ to be more affected by the interaction with the MoS_2_ ML rather than the longitudinal wave that leaks into the bulk. This reasoning is consistent with the experimental results reported in Figures 2b,c and 4a, where a main oscillatory contribution with frequency close to the longitudinal mode dominates the TG signal of samples #2 and #3, where a ML MoS_2_ is present. Considering the values of the attenuation length for Si and MoS_2_ at 39.9 nm, which are ≈237 and ≈40 nm respectively, it is evident that a MoS_2_ ML cannot significantly alter the amount of energy deposited in the Si substrates. On the other hand, the monolayer/substrate interaction leads to a thermal resistance and changes in the dynamical heat distribution and elasticity at the interface of MoS_2_/Si heterostructures; these properties are also influenced by the surface nanogroove of the substrate which is connected dynamically to the thermoelastic response.^36,37^

To give a more quantitative view, we fitted the data from samples #2 and #3 using eq 1. Besides the TG signal collected from sample #3 at the highest fluences, the resolved oscillations are limited in time to about two periods of the longitudinal mode. This does not give enough data to effectively disentangle the contributions at distinct frequencies due to the correlation among the parameters, especially for the lower fluences. Nonetheless, it is possible to give good estimates keeping some of the parameters fixed. For sample #2, fixing the frequencies and phases of the two modes from the fit to the sample #1 TG signal (Figure 2a), we obtained an average nonoscillating background decay constant τ_th_ = (170 ± 10) ps over the different positions on the sample at 0.25 mJ/cm^2^ (Figure 2c). For sample #3, we first model the response at 0.56 mJ/cm^2^ (Figure 4a), where both modes emerge, keeping all of the parameters free. The resulting frequencies are *f*_1_ = (113 ± 5) GHz and *f*_2_ = (55 ± 1) GHz, which are connected to the velocities *v*_1_ = (9.4 ± 0.4) km/s and *v*_2_ = (4.62 ± 0.09) km/s, close to the ones of the membrane. Regarding the decay constant of the acoustic modes, we obtained τ_1_ = (5 ± 1) ps and τ_2_ = (70 ± 40) ps. We then fix the frequencies, phases, and time decay constants of the acoustic modes to extract the fluence dependence of the amplitude *A*_th_ and time decay constant τ_th_, which we report in Figure 4c,d. We omit the fit results for the lowest fluence as the time decay parameter is heavily influenced by the lower signal-to-noise ratio of this trace. While *A*_th_ increases linearly in the explored fluence range, τ_th_ settles around ≈100 ps for most of the explored range. The higher value τ_th_ = (220 ± 60) ps from sample #3 at 0.23 mJ/cm^2^ may appear as an outlier, however, it is compatible with the value derived from the sample #2 data under close excitation conditions (τ_th_ = (170 ± 10) ps at 0.25 mJ/cm^2^).

**Figure 5:**
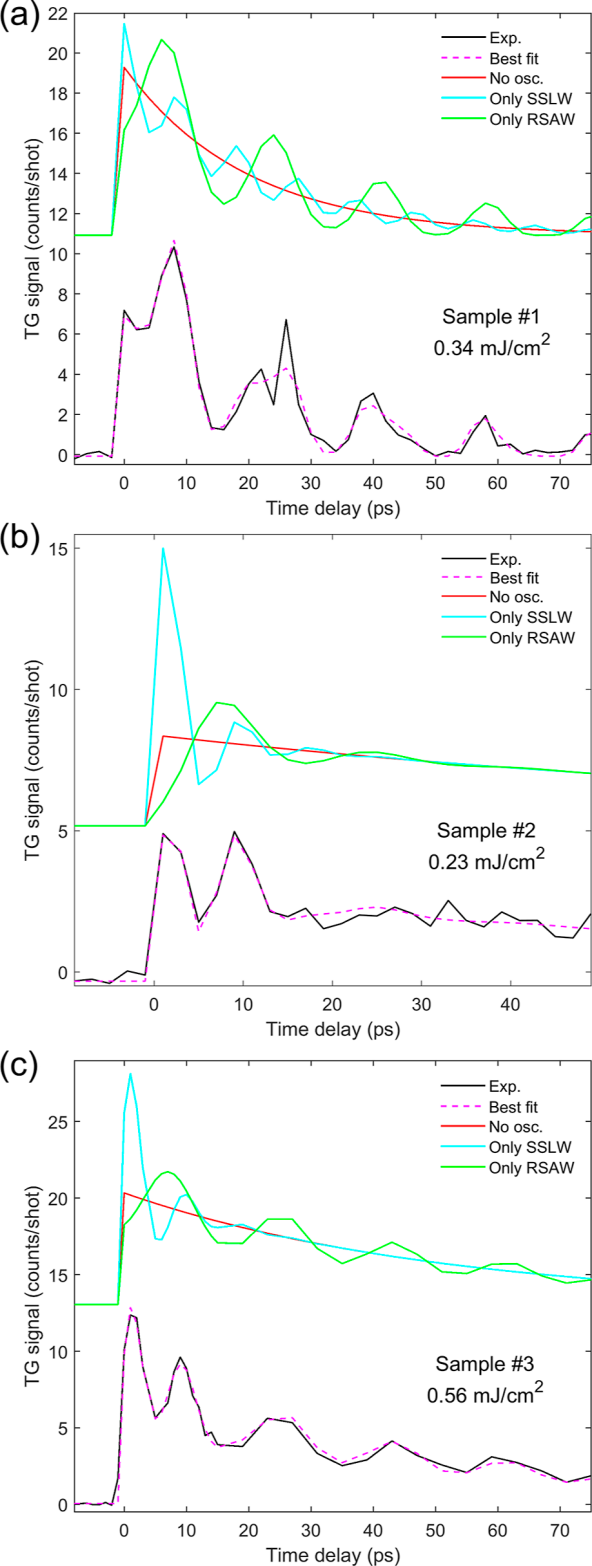
Contribution of the acoustic modes to the TG signal illustrated by comparing the experimental data (Exp.) to the best–fit curve using eq 1 (Best fit) and by selectively removing their contribution from the fit result (Only SSLW, Only RSAW and No osc., all shifted in the graph for clarity) for samples (a) #1 (b) #2 (c) #3.

To illustrate how the effects of the two acoustic modes combine differently between the blank Si membrane and the MoS_2_ heterostructures, in Figure 5 we present examples from the three samples: (a) #1 at 0.34 mJ/cm^2^, (b) #2 at 0.23 mJ/cm^2^_,_ and (c) #3 at 0.56 mJ/cm^2^. Here we remove from the fit (indicated with “Best fit”), obtained using eq 1, the contribution of one (“Only SSLW” or “Only RSAW”) or both (“No osc.”) of the two acoustic modes, by setting their amplitude to zero. It is clear that the double-peak structure found in the MoS_2_ heterostructures originates from the superposition between longitudinal and Rayleigh oscillations, where the relative height of the first two peaks is dictated by the combination of amplitude and phase of the two modes, more rapidly damped than the sample #1 case.

The observation of the influence from the ML MoS_2_ on the TG signal is favored by the choice of the substrate. We see from Figure 4a that the amplitudes of the acoustic modes increase with the pump fluence, which determines larger displacements from the atomic equilibrium positions. The attenuation length of our pump beams is ≈237 nm, comparable to the membrane thickness *b* ≈ 200 nm for sample #2. In another commonly employed substrate for TMDs such as sapphire, the attenuation length is just ≈13 nm, about 18 times smaller (see Supporting Information, Section III). This gives a much higher excitation energy density than in the case of Si, potentially giving much larger displacements due to the RSAW mode, which are localized within a depth of half of the mode wavelength in the material (Figure 3b). For fluences comparable to those employed for the data reported in Figures 2 and 4, the RSAW in the TG signal dominates the response also in a ML WS_2_/sapphire heterostructure. We provide an example of this in Supporting Information, Section V.

Finally, we discuss the decay of the nonoscillatory component of the TG signal. On sample #1, we obtained τ_th_ = (39 ± 2) ps. This value is substantially slower than what is expected by the standard thermal diffusive theory. Using the Si thermal diffusivity α_S_ ≈ 0.055 μm^2^/nS from Johnson et al.^38^ and our grating vector *k*_TG_ = 0.075 nm^–1^, the thermal diffusion theory predicts a decay τ_Si_ = 1/α_s_*k*_TG_^2^≈ 3.2 ps, about 1 order of magnitude smaller than the value from our experiment. This deviation was previously described as a consequence of ballistic transport, occurring for *k*_TG_Λ_mfp_ ≫ 1, where *k*_TG_ is the TG vector and Λ_mfp_ = 0.5–1 μm is the median phonon mean free path.^38,39^

With the addition of ML MoS_2_, we observed a marked increase in the nonoscillatory time decay in samples #2 and #3. However, the decay constant found for the heterostructure is almost as large or higher than the investigated temporal windows for samples #2 and #3 (Figures 2b,c and 4a). Therefore, a further dedicated set of measurements is required for an accurate explanation of this result. Based on the considerations for Si, it might be important to take into consideration a model that includes nondiffusive thermal transport, the interfacial thermal resistance in the MoS_2_ and Si layers^5,36^ and possibly the influence of charge recombination at the MoS_2_/Si interface.^40^ Furthermore, as we have shown in our work, the amplitude and time decay of the oscillations of the acoustic modes are markedly affected by the deposition of ML MoS_2_, suggesting a possibly less effective contribution from them to thermal transport.

## 4 Conclusions

In this work, we used nanoscale EUV transient gratings to comparatively study the nanoscale thermoelastic response of a thin Si membrane and a Si wafer, both covered with ML MoS_2_, and of a blank, thin Si membrane. The results show how a monolayer of a TMD material, in our case, MoS_2_, can dramatically modify the response of the Si substrate close to its surface. In particular, ML MoS_2_ leads to the almost complete suppression of the surface acoustic wave, which involves elliptical motion localized in the proximity of the surface, for comparable excitation fluences. Differently, the longitudinal wave, which does not involve a motion perpendicular to the surface, appears to be less affected, albeit more quickly damped as well. The two Si substrates used, a membrane and a wafer, gave a qualitatively similar behavior. Finally, we observed a slower decay of the nonoscillatory response in the heterostructure, when compared to the blank substrate. This effect is possibly connected to the impact of such acoustic modes on the transport properties of the system at the nanoscale. This work extends the time-domain investigations of the role of van der Waals materials on the acoustic response of heterostructures beyond the case of coherently excited thin layer breathing modes investigated, e.g., by time-resolved Brillouin spectroscopy.^41^ Moreover, when compared to previous TG studies on TMDs focusing on the electronic response,^15,16^ we showed that XUV TG is beneficial for understanding the interaction of monolayer–substrate interfaces. We proved that the interface strongly affects the thermoelastic properties of the bare substrate, which also provides information about the strain and stress formation in operative nanodevices. The EUV transient grating technique offers not only nanoscale sensitivity but also the necessary temporal resolution to access the dynamics of the thermoelastic response occurring on the picosecond time scale. Moreover, it is intrinsically contactless, as opposed to the need of adding metallic gratings, which would alter the morphology of the monolayer.^42^

## Supporting Information Available

Micro-Raman characterization, wavelength-dependent attenuation length of the materials, and additional transient grating data and propagation of the longitudinal and Rayleigh-like acoustic waves (PDF)
