# Supplementary material for: Impact of MoS2 Monolayers on the Thermoelastic Response of Silicon Heterostructures
Source: ACS Appl Nano Mater. 2024 Jul 1;7(13):15317–24. doi: 10.1021/acsanm.4c02096 (PMC11249974; doi:10.1021/acsanm.4c02096)
Supplement: Supplementary file 1 — an4c02096_si_001.pdf [file an4c02096_si_001.pdf]

Supporting Information on Impact of MoS_2 Monolayers on the Thermoelastic Response of Silicon Heterostructures

Davide Soranzio,[*,†] Denny Puntel,[‡] Manuel Tuniz,[‡] Paulina E. Majchrzak,[¶] Alessandra Milloch,[§,‖,⊥] Nicholas M. Olsen,[#] Wibke Bronsch,[@] Bjarke S. Jessen,[△] Danny Fainozzi,[@] Jacopo S. Pelli Cresi,[@] Dario De Angelis,[@] Laura Foglia,[@] Riccardo Mincigrucci,[@] Xiaoyang Zhu,[#] Cory R. Dean,[△] Søren Ulstrup,[¶] Francesco Banfi,[∇] Claudio Giannetti,[§,‖,††] Fulvio Parmigiani,[@,‡‡] Filippo Bencivenga,[@] and Federico Cilento[*,@]

†*Institute for Quantum Electronics, Eidgenössische Technische Hochschule (ETH) Zürich, CH-8093 Zurich, Switzerland*

‡*Dipartimento di Fisica, Università degli Studi di Trieste, IT-34127 Trieste, Italy*

¶*Department of Physics and Astronomy, Interdisciplinary Nanoscience Center (iNANO), Aarhus University, 8000 Aarhus C, Denmark*

§*Department of Mathematics and Physics, Università Cattolica del Sacro Cuore, IT-25133 Brescia, Italy*

‖*ILAMP (Interdisciplinary Laboratories for Advanced Materials Physics), Università Cattolica del Sacro Cuore, IT-25133 Brescia, Italy*

⊥*Department of Physics and Astronomy, KU Leuven, B-3001 Leuven, Belgium*

#*Department of Chemistry, Columbia University, New York, NY-10027, USA*

@*Elettra - Sincrotrone Trieste S.C.p.A., Strada Statale 14, km 163.5, IT-34149 Trieste, Italy*

△*Department of Physics, Columbia University, New York, NY 10027, USA*

∇*Université de Lyon, CNRS, Université Claude Bernard Lyon 1, Institut Lumière Matière, F-69622 Villeurbanne, France*

††*CNR-INO (National Institute of Optics), IT-25123 Brescia, Italy*

‡‡*International Faculty, University of Cologne, Albertus-Magnus-Platz, D-50923 Cologne, Germany*

E-mail: davideso@phys.ethz.ch; federico.cilento@elettra.eu

I. Micro-Raman characterization

In the main text, selected scans of the micro-Raman characterization for the Stokes scattering signal were reported in Fig. 1(b). The spectral window was centered around two peaks corresponding to the E^1_{2g} and A_{1g} near-zone center phonon modes of the MoS_2 film with frequencies at ≈385 cm^{-1} and ≈404 cm^{-1} respectively. In Fig. S1 we report, measured at the same sample positions, the peaks corresponding to the (degenerate) optical phonon mode of Si at ≈520 cm^{-1}. For sample #3, however, the Si line was saturating the detector and thus it was acquired in a separate scan with the incident power reduced by a factor 10. The Raman frequency and narrow linewidth of the Si phonon peak from the substrates confirm a single-crystal structure[1,2].

To test the uniformity of the MoS_2 film coverage for samples #2 and #3, we performed a 5x5 raster scan, *i.e.*, a series of consecutive Raman spectra taken along a regular grid.

In Fig. S2, we show the 5x5 raster scan scheme employed for the micro-Raman measurements. Panel (a) shows the coordinates of the employed grid (in µm), superimposed to sample #2 in panel (b). An equivalent pattern was used to characterize samples #1, #3 and a blank Si wafer. The spectra were fitted using a series of Voigt peaks, one for each Raman resonance on top of a quadratic background. The point by point results for the peak frequencies of the two MoS_2 Raman-active modes E^1_{2g} and A_{1g} and their separation are shown in Fig. S3. In both sample #2 and #3, we observe an overall uniform coverage with the values matching those of a monolayer film[3].

A picture of the multilayer, bulk-like, region found on the Si frame of sample #2 is reported in Fig. S4. On such island we acquired the 'bulk island' traces from Figs. 1(b) and S1.

The average frequency (f) and FWHM (w) of the MoS_2 and Si peaks for the studied samples are summarized in Tables S1, S2, together with the spectral separation of the MoS_2 peaks (Δ).

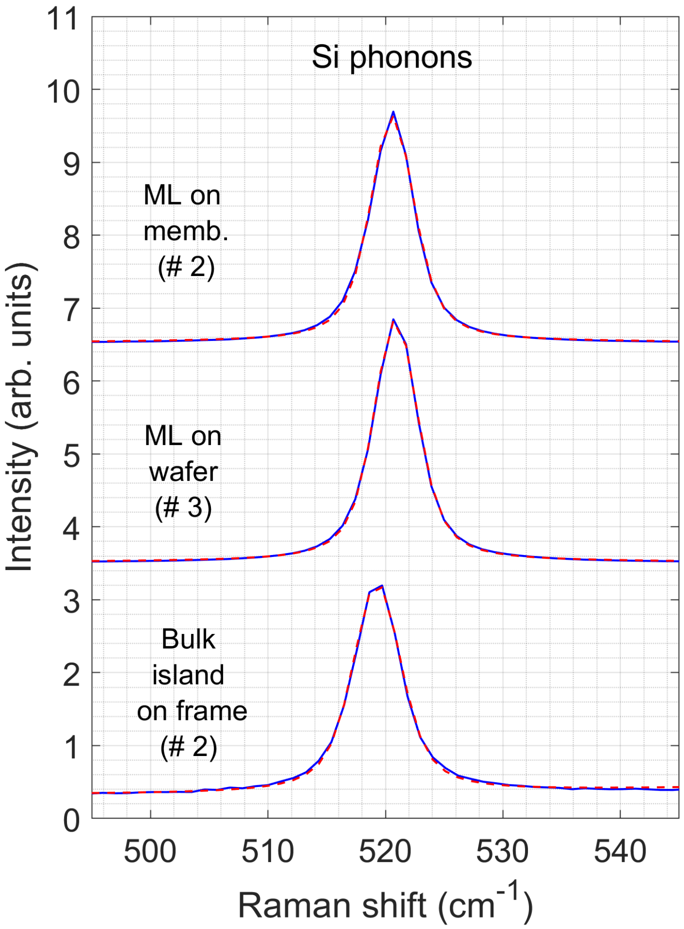

FIG. S1. Selected micro-Raman spectra showing the Si optical phonon peak from the investigated samples (MoS_2/Si membrane, MoS_2/Si wafer and a MoS_2 bulk island). The Raman traces were rescaled and vertically shifted for clarity. The blue lines correspond to the experimental data, while the red-dotted ones to the best fit to a Voigt peak on top of a quadratic background.

Table S1: Micro-Raman characterization results for regions with MoS_2 film. All the numerical results are expressed in cm^{-1}. The abbreviations 'm.', 'i.' and 'w.' stand for 'membrane', 'island' and 'wafer' respectively.

Sample	$f_{E^1_{2g}}$	$w_{E^1_{2g}}$	$f_{A_{1g}}$	$w_{A_{1g}}$	$\Delta_{A_{1g}-E^1_{2g}}$	f_{Si}	w_{Si}
MoS_2/Si m.	384.6 ± 0.1	4.02 ± 0.08	404.2 ± 0.2	6.0 ± 0.1	19.7 ± 0.3	519.97 ± 0.08	4.61 ± 0.03
MoS_2/Si i.	381.83 ± 0.04	3.83 ± 0.07	407.03 ± 0.02	4.57 ± 0.04	25.20 ± 0.05	519.29 ± 0.01	4.94 ± 0.02
MoS_2/Si w.	385.4 ± 0.1	4.26 ± 0.04	404.4 ± 0.2	6.4 ± 0.2	18.9 ± 0.4	520.91 ± 0.04	4.55 ± 0.05

Table S2: Micro-Raman characterization results for the filmless substrates. All the numerical results are expressed in cm^{-1}.

Sample	f_{Si}	w_{Si}
Si membrane	520.5 ± 0.1	4.32 ± 0.04
Si wafer	520.98 ± 0.06	4.63 ± 0.04

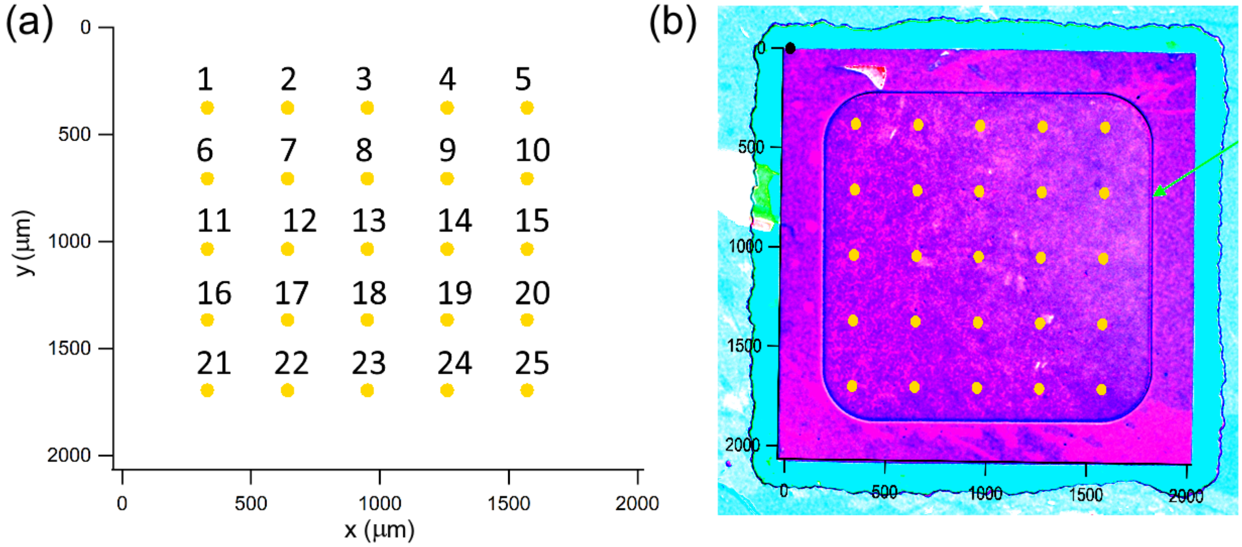

FIG. S2. (a) Scheme for the raster scan pattern to test the uniformity of the MoS_2 on the silicon substrate. The axes are in micrometers. (b) Optical-microscope picture of sample #2 after changing the tonality and saturation levels to emphasize the macroscopic morphology of the film. The light blue section corresponds to the frame, while the pink regions to the membrane. The membrane border is indicated by the green arrow. The axes and pattern from (a) are superimposed.

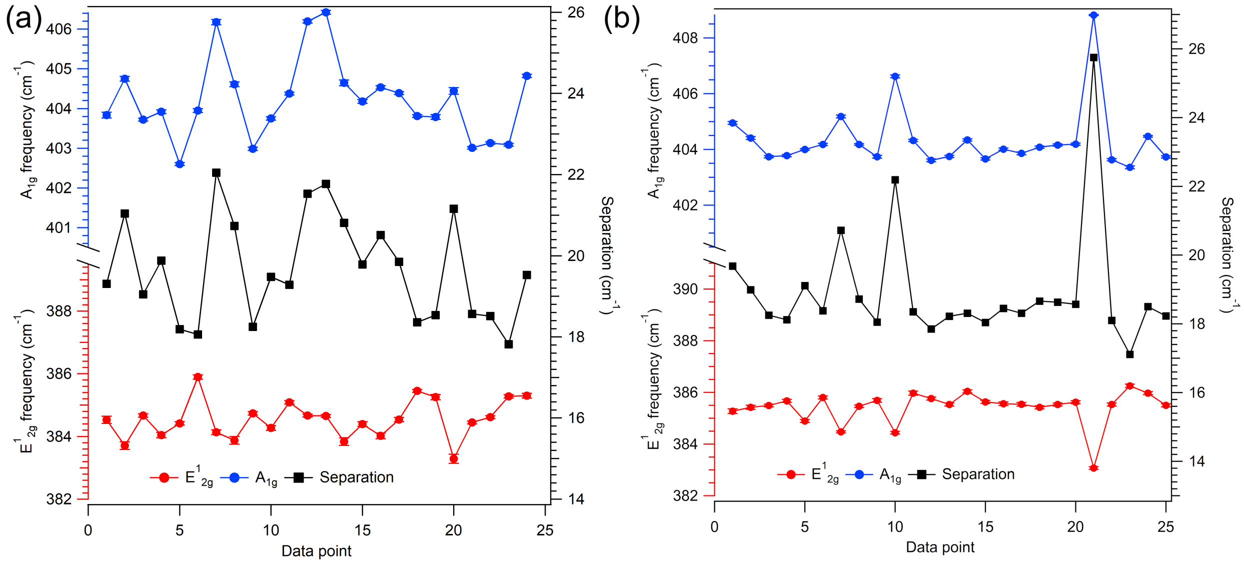

FIG. S3. Raster scan results. The left vertical scale refers to the peak positions for the E^1_{2g} and A_{1g} MoS_2 modes acquired at the positions of the raster scans (Fig. S2) through micro-Raman measurements, while the right vertical scale refers to the spectral separation between the two peaks; the linewidth of the Raman peaks was about 4 cm^{-1}. (a) Sample #2 (b) Sample #3.

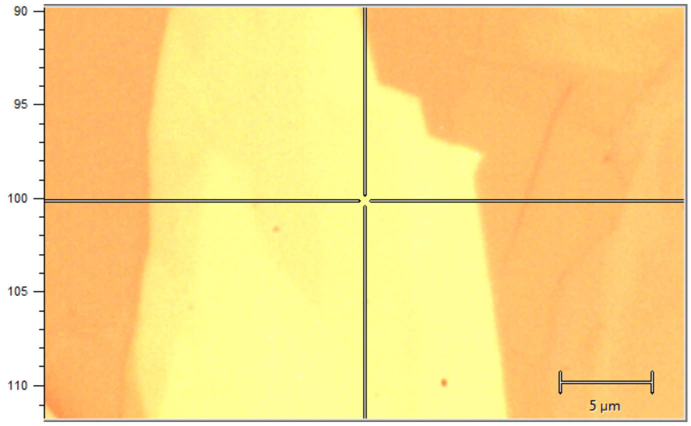

FIG. S4. Multilayer MoS_2 region found on the Si frame of sample #2, showing bulk-like response.

II. Photoluminescence measurements

While the Raman peaks reported in Figure 1(b) already indicate that the sample has a monolayer coverage, we also performed photoluminescence (PL) analysis to confirm that the sample was a monolayer. However, the PL data was quenched on the membrane. The PL spectrum on the frame region is unquenched, due to its upper thin protective SiO_2 layer, and shows that the sample is monolayer. We report an example of such data in Fig. S5.

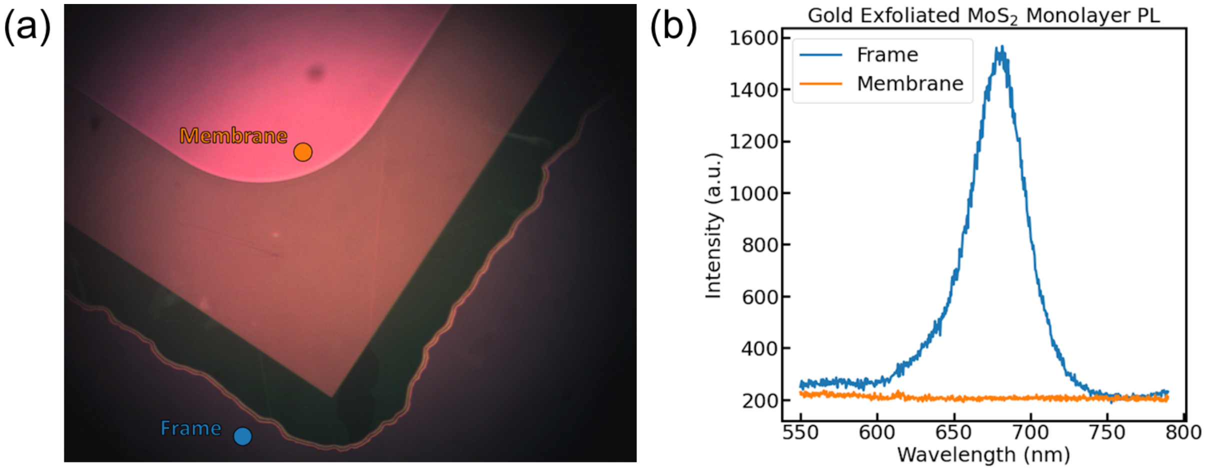

FIG. S5. Photoluminescence results. (a) Positions and (b) corresponding PL spectra for the MoS_2/Si membrane sample.

III. EUV/ Soft x-ray beam attenuation length

The EUV / Soft x-ray wavelength dependence of the attenuation length for different materials is reported in Fig. S6. As shown by the logarithmic plot, Si is the most transparent one under the wavelengths used in our transient grating FEL experiments (13.3-39.9 nm). At λ_{pu}=39.9 nm, its attenuation length (≈237 nm) is about one order of magnitude larger than the others, which indicates that the pump beams travel through the whole Si membrane used for the MoS_2/Si membrane sample (≈200 nm) without a severe energy reduction. A different case is, *e.g.*, sapphire for which the attenuation length is ≈13 nm, therefore meaning that a transient grating generated by 39.9 nm beams will be localized close to the surface and

leading to a much higher energy density close to it under the same excitation conditions.

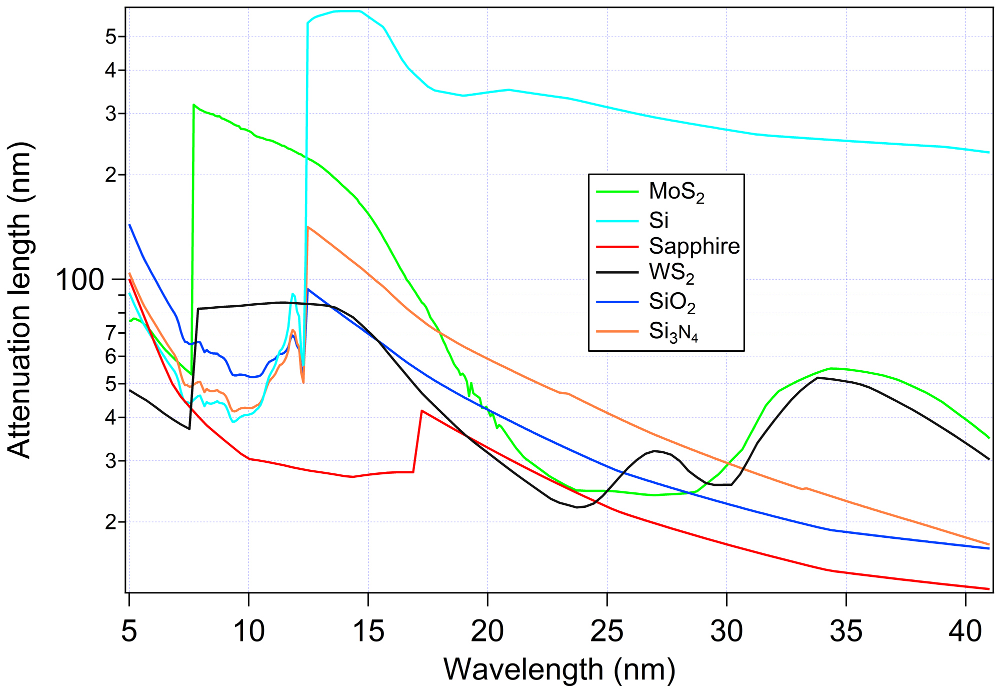

FIG. S6. EUV / Soft x-ray attenuation lengths for different materials. The curves were calculated using scattering factor data from Henke et al.[4].

IV. Acoustic waves schemes

In Figs. S7 and S8 we report simulations of the propagation of the longitudinal and Rayleigh mode in a semi-infinite bulk Si substrate at different time delays within an oscillation period. The plots were drawn using a regular 75 x 75 rectangular point grid. The displacements were calculated based on the derivation reported in Royer and Valier-Brasier using the method of potentials in the isotopic approximation (Chapter 1 and 3)[5]. The displacements $\vec{u}$ were considered at different positions x parallel to the surface and depths with respect to the surface ($y = 0$, positive values for larger depths in Si). For the longitudinal mode propagating parallel to the surface, we used a simple cosinusoidal form

$$
\begin{cases}
u_x(x,y) = U\cos(k_{TG}x - \omega t) \\
u_y(x,y) = 0
\end{cases}
\tag{1}
$$

where U is the amplitude of the mode, k_{TG} the TG wavevector and ω is the angular frequency. In order to make the shifts visible, we set the amplitude to an arbitrary value. In the plot, the color scale is normalized by the largest magnitude among the calculated displacements.

Regarding the Rayleigh wave, the displacements are

$$
\begin{cases}
Re[u_x(x,y)] = U_1(y)\sin(k_{TG}x - \omega t) \\
Re[u_y(x,y)] = U_2(y)\cos(k_{TG}x - \omega t)
\end{cases}
\tag{2}
$$

where $\omega = k_{TG}v_R$ with v_R Rayleigh velocity and the amplitudes

$$
\begin{cases}
U_1(y) = a_L\left[-k_{TG}e^{-\alpha_L y} + \sqrt{\alpha_L\alpha_T}e^{-\alpha_T y}\right] \\
U_2(y) = a_L\sqrt{\frac{\alpha_L}{\alpha_T}}\left[-\sqrt{\alpha_L\alpha_T}e^{-\alpha_L y} + k_{TG}e^{-\alpha_T y}\right]
\end{cases}
\tag{3}
$$

where a_L is the longitudinal amplitude (connected to the transverse one via $a_T = i\sqrt{\frac{\alpha_L}{\alpha_T}}a_L$), $\alpha_i = \sqrt{k_{TG}^2 - k_i^2}$ is a constant with $k_i = \omega/v_i$ connected to the velocity v_i with $i = l, t$ differentiating longitudinal and transverse parameters respectively. The longitudinal, transverse velocities and Rayleigh values were taken from Farnell and Adler[6]. To ease visibility, the amplitude a_L was set to an arbitrary value.

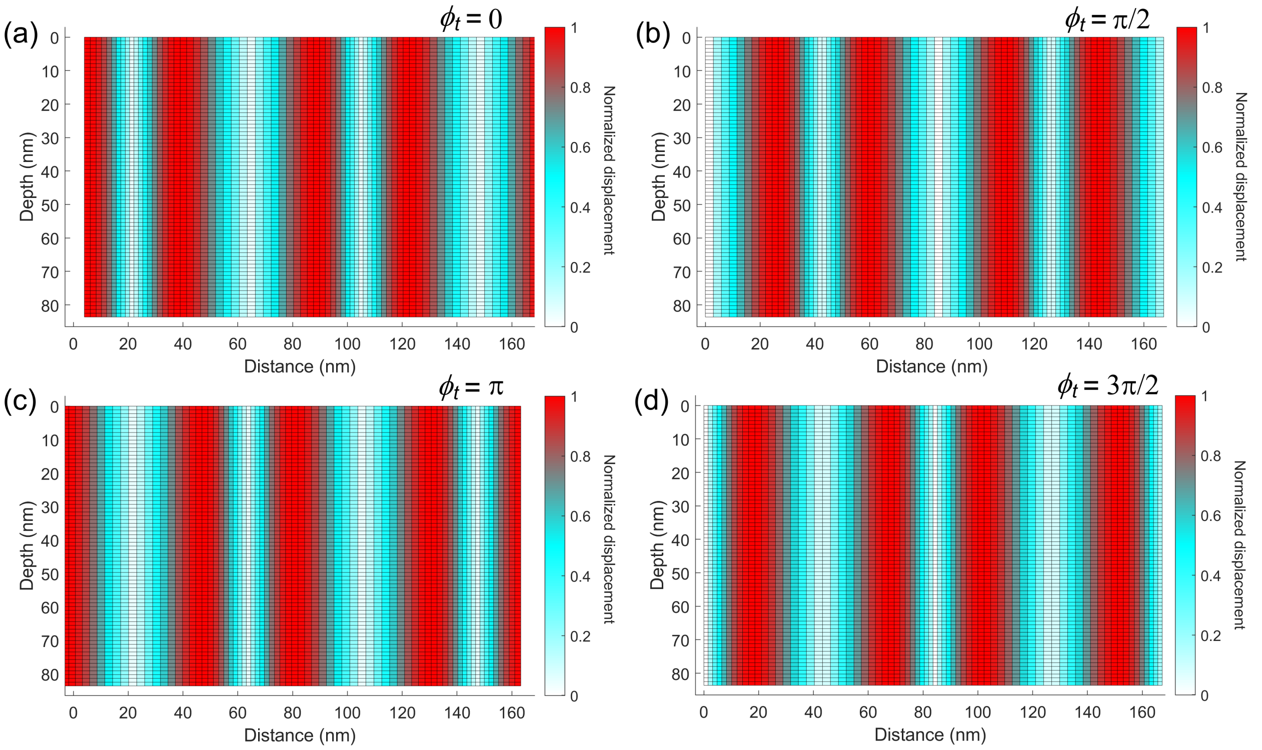

FIG. S7. Schemes of the displacements connected to a longitudinal wave in Si at four different time instants, expressed as $\phi_t = \omega t$.

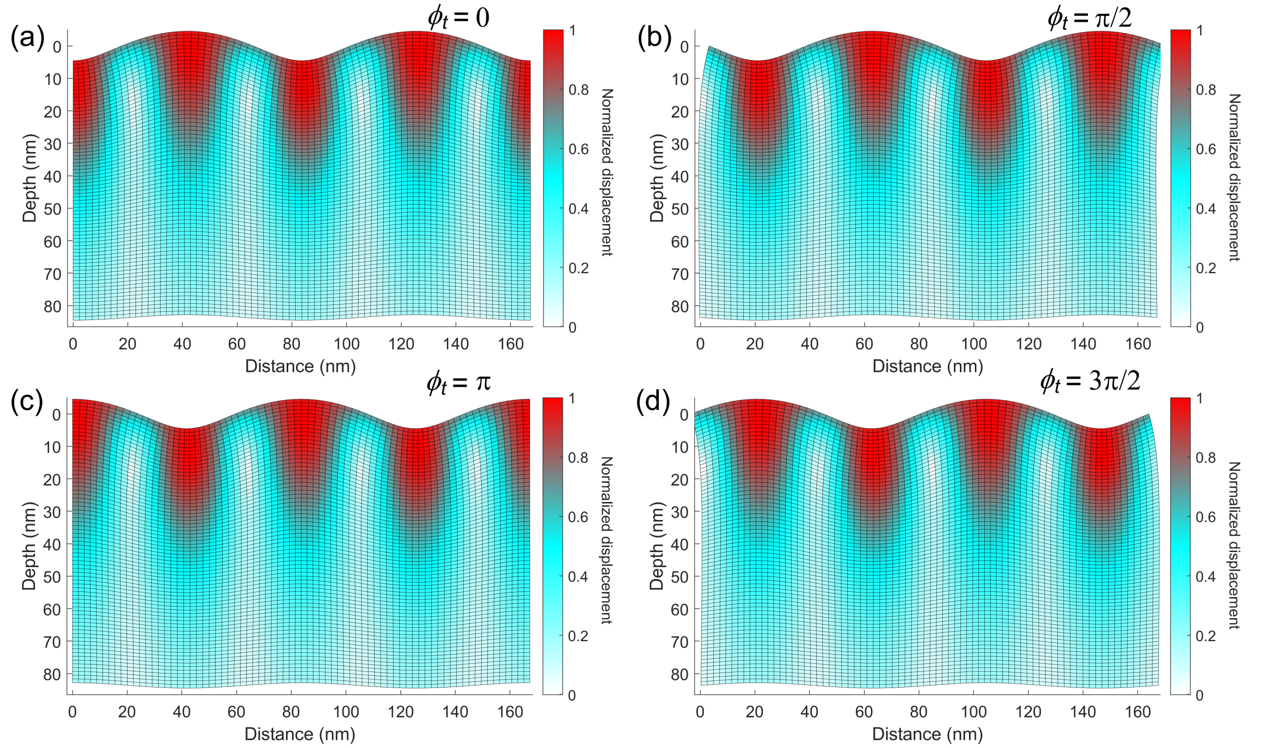

FIG. S8. Schemes of the displacements connected to a Rayleigh wave in Si at four different time instants, expressed as $\phi_t = \omega t$.

V. Additional TG results

V.I. MoS_2/Si wafer TG at different sample positions

We report in Fig. S9 the TG response collected on the MoS_2/Si wafer at different positions on the sample. While we observed some variability in the amplitude and decay time of the non-oscillating signal in the recorded window, possibly due to the local morphology of both the substrate and the film, the double-peak structure and the general characteristics of the response are maintained.

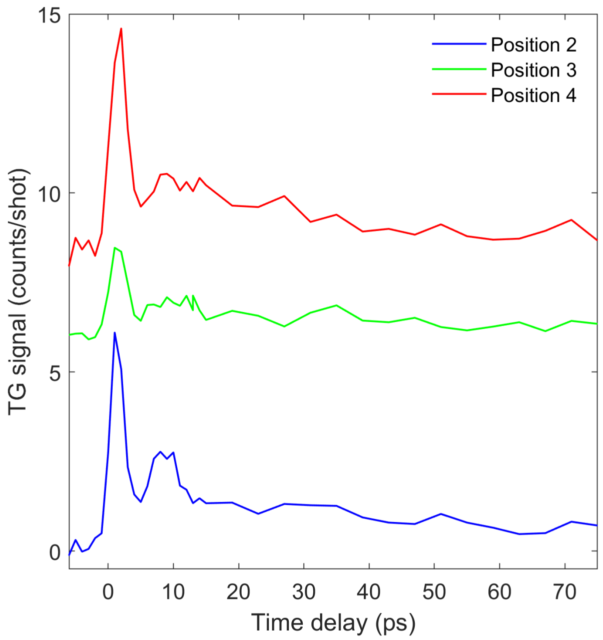

FIG. S9. TG signal from the MoS_2/Si wafer sample taken at different positions from the dataset of Fig. 4(a) at ≈0.3 mJ/cm^2 fluence.

V.II. Silicon frame

As a comparison, we show in Fig. S10 the TG signal obtained from the 200-µm-thick supporting frame of the silicon membrane. While its bulk is still made of silicon, the surface is covered by a thin oxide layer for surface protection purposes. Compared to the blank membrane response (Fig. 2(a)), the oscillatory response displays a slower decay.

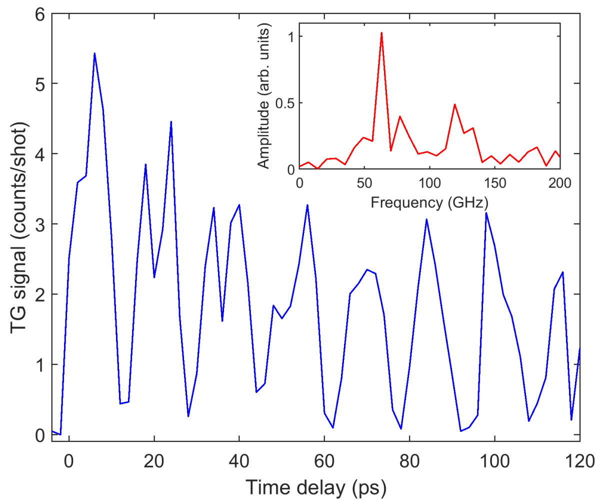

FIG. S10. Transient grating signal acquired on the Si frame of the blank Si membrane sample using a 83.6 nm grating period acquired under 0.2 mJ/cm^2 incident fluence. The inset show the discrete Fourier transform of the TG signal after removing the non-oscillatory background fitting to Eq. 1 from the main text.

V.III. WS_2/Sapphire

Fig. S11 shows in the EUV TG signal of a different heterostructure, a CVD-grown monolayer WS_2 on a ≈0.5-mm c-cut sapphire substrate (commercially available from 2D Semiconductors), collected at different grating periods, obtained by changing λ_{pu} from (a) 39.9 nm (L_{TG}=83.6 nm) to (b) 26.6 nm (L_{TG}=55.8 nm) or (c) 13.3 nm (L_{TG}=27.9 nm). Differently from the MoS_2/Si samples examined in the main text, here we notice that a single oscillatory term in Eq. 1 is enough to describe the TG response. In panel (d) we show the obtained frequencies as a function of the transient grating wavevector. Although collected at different fluences, we assume the acoustic wave propagation velocities to be almost independent from it, similarly to the MoS_2/Si wafer data in Fig. 4(a). Through a linear fit, fixing the intercept to zero due to the acoustic nature of the wave, we obtain an estimate propagation speed v=(5.398 ± 0.009) km/s. This is in good agreement with the Rayleigh mode of sapphire, reported to have a velocity ≈5.4 km/s[7]. Correspondingly, we have for the non-oscillating background decay constant τ_{th} the following fit results: (a) (38 ± 2) ps (b) (35 ± 2) ps (c) (11 ± 3) ps. The last value is, however, affected by the poorer TG signal signal-to-noise ratio

connected to the 13.3 nm pump - 13.3 probe signal, since this configuration is limited by the impossibility to freely vary the relative fluence for pump and probe. While in the second part of the explored time delay range (especially visible in panels (a) and (b)), we observe a second-harmonic modulation, connected to the square in Eq. 1, the main frequency dominates elsewhere with a possible contribution of the longitudinal-like mode the first tens of picoseconds, similarly to the samples studied in the main text. The longitudinal speed in sapphire is expected to be ≈11 km/s[8], corresponding to frequencies of (a) ≈132 GHz (b) ≈198 GHz (c) ≈395 GHz. The reason why the Rayleigh mode for this sample dominates the response even in the presence of the WS_2 film is likely connected to the much shallower penetration of the pump beams in sapphire (Fig. S6) compared to Si, leading to a significantly higher energy density near the surface, *i.e.*, where the surface acoustic waves such as the Rayleigh mode are localized. As we saw in Fig. 4(a) for the MoS_2/Si wafer sample, higher excitation energies favor the emergence of the Rayleigh mode in the TG signal in WS_2/Sapphire due to larger initial displacements.

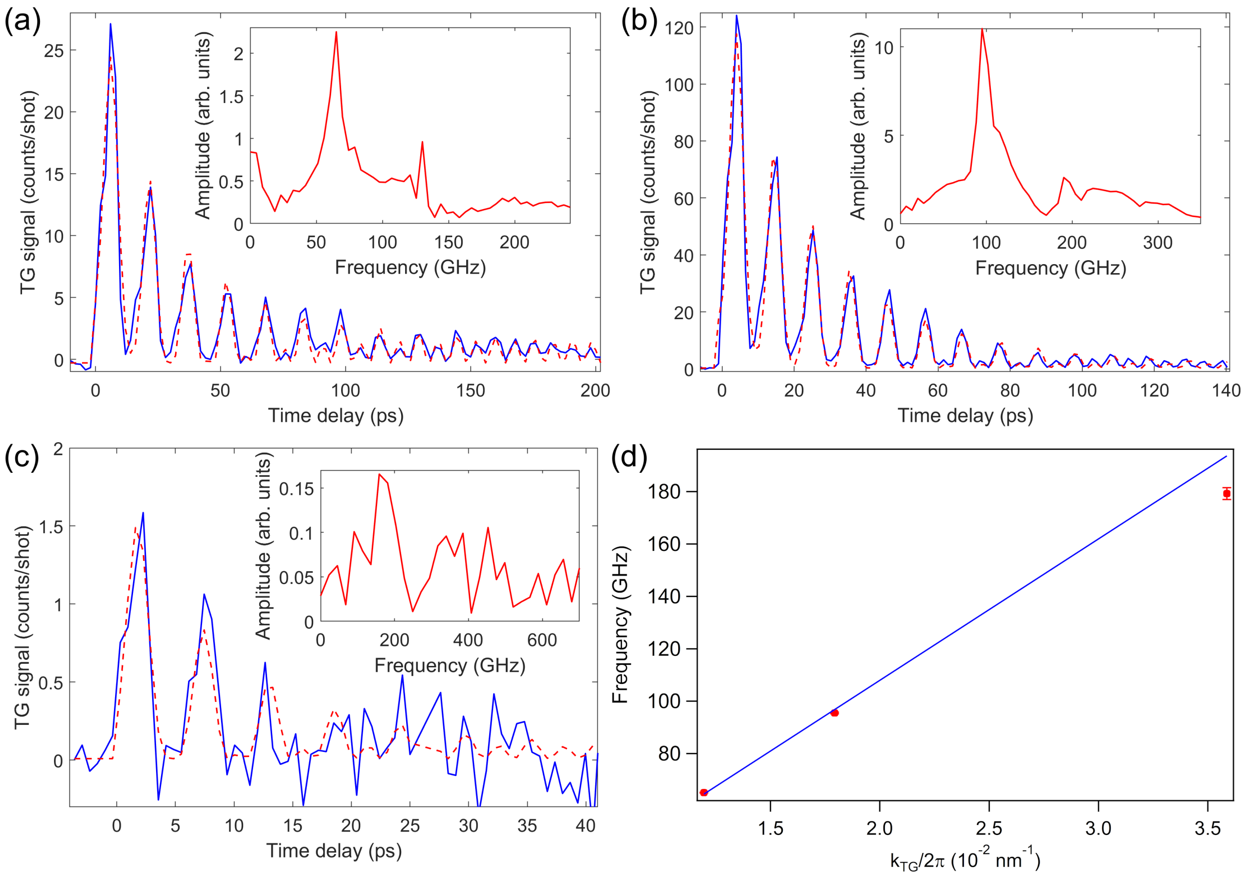

FIG. S11. TG signal for WS_2/Sapphire using different transient grating periods: (a) 84 nm under 0.1 mJ/cm^2 (b) 56 nm under 0.7 mJ/cm^2 (c) 28 nm under <0.1 mJ/cm^2 (exact estimation not accurately available). The experimental data is represented by blue traces, the fit curves to Eq. 1 (main text) by dotted red lines (d) Frequency of the Rayleigh mode as a function of the transient grating wavevector k_{TG}. The experimental data (red symbols) was fitted to a linear function, represented by the blue line, with the intercept fixed to zero.

References

(1) Lambertz, A.; Richards, B.; Sproul, A.; Puzzer, T.; Gross, M. Sputter Deposited and Solid Phase Crystallized Silicon Films for Solar Cells. Proceedings of the 2nd World Conference on Photovoltaic Solar Energy Conversion. 1998; p 1294.

(2) Yeh, W. Fabrication of single crystalline stripe in Si and Ge film on rolled flexible glass substrate by UV cw micro-chevron laser beam. Low-Dimensional Materials and Devices 2017. 2017; pp 31 – 37.

(3) Lee, C.; Yan, H.; Brus, L. E.; Heinz, T. F.; Hone, J.; Ryu, S. Anomalous Lattice Vibrations of Single- and Few-Layer MoS_2. *ACS Nano* **2010**, *4*, 2695–2700.

(4) Henke, B.; Gullikson, E.; Davis, J. X-Ray Interactions: Photoabsorption, Scattering, Transmission, and Reflection at E = 50-30,000 eV, Z = 1-92. *Atomic Data and Nuclear Data Tables* **1993**, *54*, 181–342.

(5) Royer, D.; Valier-Brasier, T. *Elastic Waves in Solids 1: Propagation*; ISTE, London and John Wiley I& Sons, New York, 2022.

(6) Farnell, G.; Adler, E. *Elastic Wave Propagation in Thin Layers*; Physical Acoustics; Academic Press, 1972; Vol. 9; pp 35–127.

(7) Pedrós, J.; Calle, F.; Grajal, J.; Jiménez Riobóo, R.; Prieto, C.; Pau, J.; Pereiro, J.; Hermann, M.; Eickhoff, M.; Bougrioua, Z. Anisotropic propagation of surface acoustic waves on nitride layers. *Superlattices and Microstructures* **2004**, *36*, 815–823, European Materials Research Society 2004, Symposium L. InN, GaN, AlN and Related Materials, their Heterostructures and Devices.

(8) Winey, J. M.; Gupta, Y. M.; Hare, D. E. r-axis sound speed and elastic properties of sapphire single crystals. *Journal of Applied Physics* **2001**, *90*, 3109–3111.
